# Validation of Wound Assessment Instruments Across Brazilian Clinical Contexts: Protocol for a Scoping Review

**DOI:** 10.2196/99483

**Published:** 2026-08-24

**Authors:** Clarissa Albuquerque Vaz Nunes, Emerson Roberto dos Santos, João Daniel de Souza Menezes, Matheus Querino da Silva, Leonila Santos de Almeida Sasso, Ana Maria Rita Pedroso Vilela Torres de Carvalho Engel, Giovana Viecili Rossi, Camila Bortoluci de Lima, Janaína Aparecida de Sales Floriano, Pedro Carlos Cagnazzo, Rauer Ferreira Franco, Alana Clara Santos Rocha, Valéria Albuquerque Vaz Rodrigues, Estella Alvarez Vieira Finoto, Cléa Dometilde Soares Rodrigues, Paula Buck de Oliveira Ruiz, Marli de Carvalho Jericó, Fabiana de Souza Orlandi, Maysa Alahmar Bianchin, Luís Cesar Fava Spessoto, Antônio Hélio Oliani, Denise Cristina Móz Vaz Oliani, Izaura dos Santos Ramos, Júlio César André, Nádia Antônia Aparecida Poletti

**Affiliations:** 1 Faculdade de Medicina de São José do Rio Preto, Center for Studies and Development of Health Education, Av. Brg. Faria Lima, 5416, São José do Rio Preto, Brazil, 55 17996140498; 2 Unifunec - Centro Universitário de Santa Fé do Sul, Santa Fé do Sul, São Paulo, Brazil; 3 Universidade Brasil, Fernandópolis, São Paulo, Brazil; 4 Expedicionários da Saúde, Campinas, Brazil; 5 Federal University of São Carlos, São Carlos, São Paulo, Brazil; 6 University of Beira Interior, Covilhã, Castelo Branco, Portugal

**Keywords:** wound assessment, content validity, expert panel, instrument validation, Brazil, scoping review protocol

## Abstract

**Background:**

Wounds are common and clinically consequential, and consistent assessment is essential for communication, monitoring, and decision-making. Although multiple wound assessment instruments exist, there is no agreement among specialists on which criteria to prioritize during validation processes and evidence synthesis remains insufficient within the Brazilian context.

**Objective:**

This scoping review aims to map which aspects of wound assessment instruments are considered important by expert health professionals when validating these instruments in the Brazilian context across clinical care settings.

**Methods:**

The review will be conducted in accordance with JBI guidance for scoping reviews. A 3-step search strategy will be used on the MEDLINE (PubMed), Embase, Scopus, Web of Science, and Cochrane Library databases, as well as in the LILACS, SciELO, and BDENF (Base de Dados em Enfermagem) databases, supplemented by gray literature sources (Brazilian Digital Library of Theses and Dissertations, CAPES Theses Catalog, and Google Scholar) and reference list screening. Records will be deduplicated and screened by 2 independent reviewers, with reporting aligned to the PRISMA-ScR (Preferred Reporting Items for Systematic Reviews and Meta-Analyses extension for Scoping Reviews) guidelines. Data will be extracted using a piloted extraction tool and synthesized descriptively in tables and with narrative mapping. Critical appraisal will not be performed.

**Results:**

This protocol was registered with the Open Science Framework on February 6, 2026. Database searches are scheduled to commence following protocol publication. Data collection is projected to begin in July 2026, with screening and data extraction expected to be completed by December 2026, and the final review report planned to be submitted for publication in the first quarter of 2027. Results will map the extent, nature, and distribution of evidence on expert-led validation of Brazilian wound assessment instruments across clinical settings.

**Conclusions:**

This scoping review will provide a systematic and transparent synthesis of which aspects health professionals consider when validating Brazilian national wound assessment instruments. We expect the findings to identify methodological gaps, inform future validation studies, and support the development and refinement of nationally applicable instruments across diverse Brazilian health care contexts.

## Introduction

Acute and chronic wounds represent a significant public health challenge in Brazil, affecting millions of individuals and imposing a substantial burden on patients and the health care system. A multicenter study conducted in primary care settings across 5 Brazilian regions reported a prevalence of 4.3% among adults, with higher rates among older adults, individuals with diabetes, and those with cardiovascular disease [1]. The associated direct costs, including dressings, hospitalizations, and professional time, and indirect costs, such as functional loss and reduced quality of life, position wound care among the most resource-intensive conditions across all levels of care [2-5]. Wound healing is a dynamic, multifactorial process in which systematic assessment of the lesion, dimensions, wound bed tissue, exudate, wound edges, periwound skin, signs of infection, and temporal evolution is central to therapeutic decision-making and outcome monitoring [6,7]. Nevertheless, in many services, wound assessment remains dependent on subjective judgment and heterogeneous records, limiting case comparability and hindering care standardization. Evidence also indicates gaps in assessment knowledge and competence among nursing professionals, with repercussions for delayed healing and suboptimal therapeutic choices [8,9]. Structured wound assessment instruments are therefore strategic for reducing variability, supporting clinical decisions, and improving care quality [7,10].

Reviews suggest that many existing instruments emphasize monitoring healing trajectories while there remains demand for tools that also strengthen clinical reasoning about etiology and complexity [7]. Applicability further depends on cultural adaptation, operational clarity, and adequate training. A Brazilian integrative review found that instruments commonly assess size, depth, exudate, tissue type, and infection signs, yet it underscored the need for measures supported by robust evidence [11]. Cardinelli et al [12] mapped 51 wound assessment instruments and noted that only a small proportion had undergone validation or cross-cultural adaptation for the Brazilian context. Lima and Paes [13] identified methodological diversity and lack of consensus in tools for wound infection assessment; Almeida et al [14] emphasized the scarcity of studies evaluating clinical applicability and validity for chronic lower-limb wounds in national contexts [3]. Collectively, these findings reveal a practical problem: even when tools exist, evidence is often insufficient to demonstrate their validity, feasibility, and usefulness in Brazilian real-world care.

Instrument validation represents the inflection point between having a form and having a reliable, valid measure. Validation involves conceptual development, content validity assessment via expert panels, reliability testing, and analysis of measurement properties [15-17]. At the core of content validity, experts evaluate items for relevance, clarity, representativeness, and operational adequacy; methods such as the content validity index (CVI) are commonly used but vary in criteria, number of judges, and cutoffs, introducing heterogeneity and controversy [18-20]. Despite the growth of publications on wound instruments, the specific aspects considered by experts during validation of Brazilian national instruments remain insufficiently systematized [21]. It is still not clear which criteria specialists prioritize (eg, clinical relevance, semantic clarity, applicability across settings, responsiveness, and compatibility with documentation routines), or how these criteria are operationalized in validation studies. This gap is particularly relevant because instruments with inadequate or poorly transparent validation may produce superficial standardization without ensuring interrater consistency or supporting safe clinical decision-making.

A scoping review is the most appropriate design to address this gap. Unlike systematic reviews, which synthesize evidence to answer a specific clinical question through formal critical appraisal, scoping reviews map the extent, range, and nature of available evidence, making them suitable when the field is heterogeneous and the objective is to identify gaps rather than estimate effects [22-26]. Compared with integrative reviews, they offer a broader, more exploratory approach without hierarchical weighting of sources [23,27]. JBI methodology and the PRISMA-ScR (Preferred Reporting Items for Systematic Reviews and Meta-Analyses extension for Scoping Reviews) extension explicitly support this design when the objective is to describe the breadth of evidence and clarify definitions [23,27-29]. This protocol therefore proposes a scoping review without temporal restriction, including evidence in Portuguese, English, and Spanish, to answer the following review question: Which aspects are considered by expert health professionals during the validation process of Brazilian national instruments intended for wound assessment?

## Methods

### Overview

This scoping review will be conducted in accordance with the JBI methodology for scoping reviews [28], as recommended in the JBI Protocol Template for Scoping Reviews (2024). The present protocol report follows the JBI 2022 reporting items for scoping review protocols [30], and the completed scoping review will be reported in accordance with the PRISMA-ScR checklist [31]. A 3-step search strategy will be applied in the MEDLINE (PubMed), Embase, Scopus, Web of Science, and Cochrane Library databases, as well as in the LILACS, SciELO, and BDENF (Base de Dados em Enfermagem) databases, supplemented by gray literature sources and reference list screening. Records will be deduplicated and screened by 2 independent reviewers, with reporting aligned to PRISMA-ScR. Data will be extracted using a piloted extraction tool and synthesized descriptively using tabular presentation and narrative mapping. In accordance with JBI guidance for scoping reviews [28], critical appraisal will not be performed, as discussed in the Critical Appraisal section.

### Inclusion Criteria

#### Participants

Studies will be included if they involve expert health professionals in wound management and assessment, such as nurses, physicians, and other health professionals with recognized expertise in wound care. Participants must have documented experience relevant to validation and/or expert use of wound assessment instruments and must act as evaluators, judges, or validators in the study. Evidence sources that do not involve these professionals in such roles will be excluded to preserve focus on specialized expert judgment.

#### Concept

Evidence sources addressing validation of Brazilian national wound assessment instruments will be considered. Validation may include, but is not limited to, content validity, reliability, applicability, feasibility, and clarity and interpretability of the instrument. Consistent with the COSMIN (Consensus-Based Standards for the Selection of Health Measurement Instruments) taxonomy of measurement properties [16], validation in this review encompasses content validity (including expert assessment of relevance, comprehensiveness, and comprehensibility), reliability, criterion validity, construct validity, and responsiveness. To align with the review question, only studies in which expert health professionals explicitly participate as evaluators, judges, or validators in at least one validation stage will be included. Studies addressing only measurement properties that do not involve expert judgment (eg, interrater reliability without content evaluation) will be excluded, as they do not inform the aspects prioritized by specialists. Instruments assessing wounds in different clinical contexts will be eligible, provided that the validation is explicitly national (ie, developed in Brazil or culturally adapted and validated for Brazilian use). Studies addressing international instruments without Brazilian validation or those describing wound assessment without methodological focus on validation will be excluded. It should be noted that “applicability/feasibility” and “clarity/interpretability” are not standard COSMIN properties; where reported in included studies, these will be mapped to the relevant COSMIN domains of content validity (comprehensibility) and interpretability, respectively.

#### Context

The review will include studies conducted within the Brazilian context, acknowledging cultural, organizational, political, and geographic factors that may influence the development and validation of instruments. Diverse health care settings will be eligible, including primary, secondary, and tertiary care settings, provided that they relate to Brazilian populations and practice. Studies developed outside Brazil without analysis or cultural adaptation for the national context will be excluded.

### Types of Sources

This scoping review will consider a wide range of study designs and evidence types to provide a comprehensive map of available evidence on validation of Brazilian wound assessment instruments involving expert judges and validators. Eligible sources will include experimental and quasi-experimental studies (eg, randomized and nonrandomized trials, before-and-after studies, and interrupted time series), analytical observational studies (eg, prospective and retrospective cohort studies, case-control studies, and analytical cross-sectional studies), and descriptive observational studies (eg, case series, case reports, and descriptive cross-sectional studies), when they report validation-related aspects. Experimental and observational studies contribute evidence on the applicability and measurement properties of instruments across clinical populations. Descriptive studies provide insights into the operationalization of validation procedures in real-world Brazilian settings.

Qualitative studies exploring the perspectives and participation of experts in instrument development and validation (eg, phenomenology, grounded theory, ethnography, qualitative descriptive research, and action research) will also be included to capture experiential and conceptual criteria used by specialists. Qualitative studies are relevant because they can reveal tacit criteria, decision-making processes, and contextual factors that influence expert judgment during validation. Systematic reviews meeting the inclusion criteria, as well as relevant text and opinion papers, will be considered to broaden conceptual understanding. Systematic reviews synthesize prior evidence on validation practices and may identify methodological recommendations; opinion papers and technical documents offer expert perspectives and practical frameworks that inform how validation criteria are operationalized. To ensure relevance and breadth, peer-reviewed publications, academic works (theses and dissertations), technical documents, and pertinent gray literature will be eligible. Trials, case reports, and case series will be included only when they explicitly describe expert involvement in a validation stage of a wound assessment instrument; evidence sources that do not directly report such expert participation will be excluded. Gray literature (theses, dissertations, technical reports, and conference proceedings) is included to capture validation studies that may not have been published in peer-reviewed journals but contain detailed descriptions of expert panel methodologies.

### Search Strategy

The search strategy will aim to locate both published and unpublished studies and will follow the 3-step approach recommended by JBI. Step 1 will comprise an initial limited search in PubMed and Scopus to identify relevant articles. Text words in titles and abstracts of relevant papers, and the index terms used to describe those papers, will be used to develop a comprehensive search strategy for each included database. Step 2 will involve executing the full search strategy in the MEDLINE (PubMed), Embase, Scopus, Web of Science, and Cochrane Library databases, as well as in the LILACS, SciELO, and BDENF databases, adapting syntax and controlled vocabulary to each source. For the LILACS, SciELO, and BDENF databases, parallel search blocks will be developed in Portuguese using DeCS (Descritores em Ciências da Saúde) descriptors, combined with the same conceptual blocks (instruments, wounds, and expert professionals). The complete Portuguese search strategy is presented in Multimedia Appendix 1. Step 3 will consist of screening reference lists of included sources to identify additional eligible evidence.

The detailed search strategies for each database are presented in Multimedia Appendix 1. Strategies combine terms related to instruments, scales, classification, or stratification tools and assessment of skin integrity or cutaneous lesions (eg, the Bates-Jensen Wound Assessment Tool [BWAT], Resultados en la Valoración y Evolución de la Cicatrización de las Heridas [RESVECH], Pressure Ulcer Scale for Healing [PUSH], site, ischemia, neuropathy, bacterial infection, area, and depth [SINBAD], Depth, Exudate, Size, Inflammation/Infection, Granulation tissue, Necrotic tissue, Pocket Rating [DESIGN-R], and Freiburg Life Quality Assessment for Wounds [FLQA-Wk], as well as generic terms such as *wound assessment instrument*, *wound healing scale*, and *wound classification system*) and methodological terms (eg, *validation*, *psychometrics*, *content validity*, *reliability*, *cross-cultural adaptation*, and *measurement properties*), terms related to wounds or skin lesions (eg, *wounds*, *pressure injuries*, and *chronic wounds*), and terms related to experts and professionals (eg, *specialists*, *experts*, and *wound specialists*), according to each database’s specific syntax. Methodological terms such as *validation*, *psychometrics*, *content validity*, *reliability*, and *cross-cultural adaptation* will be incorporated into the instrument or methodology conceptual block to ensure that studies using formal validation approaches are captured even when specific instrument names do not appear in the title or abstract. Per-database search results will be recorded and reported separately in the final review.

No language or time restrictions will be applied. Evidence sources published in Portuguese, English, or Spanish will be included. No temporal restriction will be applied. The year of publication will be recorded as a descriptive variable and reported in the final review to allow readers to assess the temporal distribution of the evidence. This approach avoids excluding methodologically relevant studies, such as the Brazilian adaptation of the BWAT [32], earlier cross-cultural adaptations of the FLQA-Wk, and the foundational mapping by Cardinelli et al [12], which predate 2020 but remain pertinent to the review question. The absence of a date limit is consistent with JBI guidance for scoping reviews, which recommends restricting by date only when justified by the review objective [28].

Gray literature sources will include Google Scholar (first 200 records sorted by relevance), the Brazilian Digital Library of Theses and Dissertations (BDTD), and the CAPES Theses and Dissertations Catalog, as well as institutional websites, technical documents, conference proceedings, and, when appropriate, direct contact with authors. Gray literature records will be screened following the same process involving 2 independent reviewers described for database records. The number of gray literature sources identified, screened, and included will be tracked separately and reported in the PRISMA-ScR flow diagram.

### Study or Source of Evidence Selection

Following the searches described above and in Multimedia Appendix 1, all identified citations will be compiled and imported into Rayyan for screening management and study selection. Duplicates will be identified and removed prior to screening.

Before formal screening, a pilot test will be undertaken using a sample of records to calibrate consistent application of the inclusion and exclusion criteria. Title and abstract screening will be performed independently by 2 reviewers (JDdSM and MQdS). Potentially eligible sources will be retrieved in full text and assessed in detail by the same two independent reviewers (JDdSM and MQdS) for final inclusion. Disagreements at any stage will be resolved through discussion and consensus; if required, a third reviewer (JCA) will adjudicate.

Reasons for exclusion at the full-text stage will be recorded and reported in the final scoping review manuscript.

The search and selection process will be fully described in the final report and presented using a PRISMA (Preferred Reporting Items for Systematic Reviews and Meta-Analyses) flow diagram (preferably PRISMA-ScR), indicating the numbers of records identified, screened, assessed for eligibility, excluded (with reasons), and included.

### Data Extraction

Data will be extracted from included sources of evidence by 2 independent reviewers (JDdSM and ERdS) using a data extraction tool developed by the review team, as recommended in the JBI Protocol Template for Scoping Reviews (2024). The complete extraction instrument is provided in Multimedia Appendix 2.

The adjudication structure follows a 2-tier approach: (1) initial resolution through discussion and consensus between the two primary reviewers at each stage, and (2) if consensus cannot be reached, independent adjudication by a senior reviewer (JCA), whose decision will be final and recorded. This structure applies uniformly to title and abstract screening, full-text eligibility assessment, and data extraction.

Before large-scale extraction begins, the form will be piloted on a sample of included studies to ensure clarity and consistency between reviewers (calibration). The instrument may be revised during the extraction process as necessary; any modifications will be recorded and described in the final report.

Information to be extracted will include the following, at minimum: (1) source identification (study ID or code, authors, year, title, journal or source, DOI or link, country, publication type, funding or conflict of interest statements); (2) source characteristics (objective, study type or methodology, participants, clinical context, target population of the instrument, instrument assessed, validation stages reported such as content validity, criterion validity, reliability, responsiveness, and the analytical and statistical methods used, eg, Cronbach α, κ, and intraclass correlation coefficient); (3) results and evidence (main findings, psychometric indicators, barriers, suggested improvements, applicability across contexts, cultural or linguistic adaptation); and (4) gaps and limitations. Additionally, the following fields have been added to the extraction tool (Multimedia Appendix 2): validation framework used (eg, COSMIN), number of experts involved, and expert eligibility criteria reported.

Data extraction may be operationalized using a spreadsheet (eg, Microsoft Excel or Google Forms) or directly within review support software (eg, Rayyan), preserving editable fields and space for reviewer comments. Discrepancies between reviewers will be resolved through discussion and, if necessary, consultation with an additional reviewer. When appropriate, study authors may be contacted to request missing information or additional data.

### Critical Appraisal

No critical appraisal of included sources will be undertaken, consistent with the purpose of a scoping review to map the extent and nature of evidence. This decision follows the JBI 2022 guidance for scoping reviews, which states that critical appraisal is optional in scoping reviews and should be justified based on the review objective [28]. Given that this review aims to identify and map the aspects considered by experts during validation, rather than to synthesize evidence on the effectiveness or quality of specific instruments, formal risk-of-bias assessment is not required. However, as recommended by JBI [28], a brief descriptive characterization of the methodological frameworks used in the included studies (eg, COSMIN, Beaton, Pasquali, or Polit & Beck) will be provided in tabular format to allow readers to assess the rigor of the reported validation processes.

### Data Analysis and Presentation

Data analysis and presentation will be conducted to respond directly to the objectives and review question. Extracted data will be organized and mapped predominantly in tabular format and, where appropriate, in diagrammatic or graphic form (eg, descriptive charts and flow diagrams), consistent with JBI guidance.

Results will be synthesized using simple descriptive statistics (frequencies and proportions), without meta-analysis, and presented in tables and figures that allow visualization of: (1) characteristics of included sources (author, year, country, publication type, and journal or source), (2) instruments assessed and their characteristics, (3) Brazilian clinical contexts in which instruments were applied or tested (eg, hospital, primary care, outpatient, and home), (4) target populations (eg, adults, older adults, and chronic wounds), (5) validation stages and evidence reported (eg, content validity, criterion validity, reliability, and responsiveness), (6) psychometric metrics reported (eg, Cronbach α, kappa, and intraclass correlation coefficient), and (7) expert involvement (presence or absence, number, and profile), aligned with the extraction tool in Multimedia Appendix 2.

In addition, a narrative and visual mapping of gaps will be produced (eg, methodological limitations, omission of validation steps, barriers to applicability, and needs for cultural or linguistic adaptation). A narrative synthesis will accompany tabulated and/or graphical results, describing how findings relate to the objectives and question, and highlighting patterns, convergences, inconsistencies, and areas with insufficient evidence.

### Consultation With Experts

Following preliminary synthesis, a consultation phase will be undertaken to refine and contextualize the findings of the scoping review. Preliminary results will be presented to a group of health professionals with recognized expertise in instrument validation and/or wound assessment in Brazil. Experts will be invited to provide feedback on the clarity, relevance, and comprehensiveness of the mapped aspects; to support interpretation; and to discuss implications for practice and future research. The consultation process will be conducted using semistructured interviews, and feedback will be incorporated into the final report to enhance ecological validity and applicability. As this phase involves primary data collection from human participants, ethics approval will be sought from the Research Ethics Committee (Comite de Etica em Pesquisa and CONEP) prior to its initiation.

## Results

### Overview

This is a protocol article describing the planned methodology for the scoping review. As of July 2026, the protocol has been registered with the Open Science Framework on February 6, 2026 (3FSTE). Database searches are scheduled to commence following protocol publication. Funding for this project was approved by the National Council for Scientific and Technological Development (CNPq), Brazil, in 2025. Data collection is projected to begin in July 2026, with screening and data extraction expected to be completed by December 2026, and the final review report submitted for publication in the first quarter of 2027.

The PRISMA-ScR flow diagram will be presented in the full review report, detailing the number of records identified, screened, assessed for eligibility, excluded (with reasons), and included at each stage of selection. The complete data extraction tables, as specified in Multimedia Appendix 2, will be provided in the final manuscript. The PRISMA-ScR checklist will be included as a multimedia appendix to the final review report.

### Timeline

A tentative timeline for conducting the scoping review is presented in Table 1. This timeline may be adjusted depending on resource availability and the volume of evidence identified.

**Table 1.** Tentative timeline for scoping review conduct.

Stages	Estimated duration
Protocol registration	1 week
Database searches	2 weeks
Deduplication and screening (title and abstract)	4 weeks
Full-text retrieval and selection	6 weeks
Data extraction	8 weeks
Data analysis and synthesis	4 weeks
Consultation with experts	3 weeks
Final report writing	6 weeks
Submission for publication	2 weeks

## Discussion

### Anticipated Findings

This scoping review is expected to produce a comprehensive map of the aspects considered by expert health professionals during the validation of Brazilian national wound assessment instruments. The anticipated findings will describe the range of validation stages addressed in the literature (eg, content validity, reliability, and criterion validity), the profile of expert judges involved, the clinical settings and target populations covered, and the psychometric approaches used. Rather than testing a hypothesis, this map will identify patterns, methodological convergences, and evidentiary gaps across the Brazilian wound care literature.

Previous Brazilian reviews have mapped wound assessment instruments and their general measurement domains [11,12], assessed tools for wound infection [13], and examined instruments for chronic lower-limb wounds [14]. Cardinelli et al [12] identified 51 instruments but noted that only a small proportion had undergone Brazilian validation. The present review extends this body of work by shifting the analytical focus from the instruments themselves to the validation process, specifically, the aspects prioritized by expert judges. This perspective is novel because it addresses the operational criteria, methodological frameworks, and reporting practices that underlie content validity and expert judgment in the Brazilian context. Internationally, the COSMIN initiative [16] and Beaton et al [15] have established standards for cross-cultural adaptation and measurement property assessment; however, no prior review has systematically examined how these standards are operationalized in Brazilian wound care validation studies or which aspects experts consider most relevant.

The main strengths of this protocol include its adherence to JBI methodology [28], registration with the Open Science Framework, a comprehensive multidatabase search strategy incorporating Brazilian and Latin American databases (LILACS, SciELO, and BDENF) with parallel Portuguese search blocks using DeCS descriptors, systematic gray literature searches through BDTD and the CAPES Catalog, the absence of a temporal restriction to capture methodologically relevant studies regardless of publication year, and a consultation phase with experts to enhance the ecological validity of the findings. Limitations include the inherent heterogeneity in definitions and reporting of validation steps across included studies, which may complicate synthesis. Despite systematic searches in gray literature repositories, some relevant theses, technical documents, or institutional reports may remain inaccessible. The language restriction to Portuguese, English, and Spanish, while appropriate for the Brazilian focus, may exclude relevant studies published in other languages. The absence of critical appraisal, although justified per JBI guidance for scoping reviews [28], means that the resulting map will not include a formal assessment of the methodological quality of individual studies. The consultation phase with experts is planned to partially mitigate interpretive limitations and enhance the practical relevance of the findings.

The findings of this review are expected to inform several lines of future work. First, they may support the development of a national methodological guideline for wound instrument validation in Brazil, addressing identified gaps in expert selection criteria, CVI threshold standards, and reporting completeness. Second, the map of underresearched clinical settings and populations may guide prioritization of future validation studies. Third, the review may identify instruments that require updated or expanded validation before they can be recommended for widespread clinical use, and may highlight opportunities for cross-cultural adaptation of instruments that have been validated in other contexts but lack Brazilian evidence.

The completed scoping review will be submitted for publication to a peer-reviewed journal. The PRISMA-ScR checklist will be included as a multimedia appendix to the final report. The findings will also be presented at national conferences on wound care and stomal therapy, and shared with collaborating professional societies, including the Brazilian Society of Dermatology Nursing and the Brazilian Society of Enterostomal Therapy, to maximize their reach to clinicians and researchers involved in wound assessment instrument validation.

### Conclusions

The scoping review described in this protocol is distinctive in its methodological scope: it incorporates Brazilian and Latin American databases (LILACS, SciELO, and BDENF) with parallel Portuguese-language search blocks using DeCS descriptors, includes systematic gray literature searches through BDTD and the CAPES Catalog, applies no temporal restriction to capture methodologically relevant studies irrespective of publication year, and integrates a consultation phase with experts to enhance the ecological validity of the synthesized findings. The resulting evidence map is expected to clarify the operational criteria currently used in expert validation of Brazilian wound assessment instruments, identify areas of methodological convergence and inconsistency across studies, and support the development of evidence-informed guidelines for instrument validation in the national context. By mapping what aspects experts prioritize and how these are operationalized across clinical settings, this review may contribute to reducing heterogeneity in validation practices, strengthening the methodological basis for future instrument development, and ultimately supporting more consistent, high-quality wound assessment across Brazilian health care.

## Supplementary material

10.2196/99483 Multimedia Appendix 1 Search strategies for all databases searched.

10.2196/99483 Multimedia Appendix 2 Data extraction instrument for the scoping review.

## Abbreviations

BDENF: Base de Dados em Enfermagem
BDTD: Brazilian Digital Library of Theses and Dissertations
BWAT: Bates-Jensen Wound Assessment Tool
CNPq: National Council for Scientific and Technological Development
COSMIN: Consensus-Based Standards for the Selection of Health Measurement Instruments
CVI: content validity index
DeCS: *Descritores em Ciências da Saúde*
FLQA-Wk: Freiburg Life Quality Assessment for Wounds
PRISMA: Preferred Reporting Items for Systematic Reviews and Meta-Analyses
PRISMA-ScR: Preferred Reporting Items for Systematic Reviews and Meta-Analyses extension for Scoping Reviews
