# Supplementary material for: Validation of Wound Assessment Instruments Across Brazilian Clinical Contexts: Protocol for a Scoping Review
Source: JMIR Res Protoc. 2026 Aug 24;15:e99483. doi: 10.2196/99483 (PMC13502934; doi:10.2196/99483)
Supplement: Supplementary file 1 [file resprot-v15-e99483-s001.docx]

**Multimedia Appendix 1.** Search Strategies for All Databases Searched

**Database search strategies.**

| **Database** | **Search Strategy** |
| --- | --- |
| PubMed | ("BWAT" OR "Bates-Jensen Wound Assessment Tool" OR "RESVECH" OR "RESVECH 2.0" OR "PUSH" OR "Pressure Ulcer Scale for Healing" OR "PUSH Tool" 3.0 OR "SINBAD" OR "DESIGN-R" OR "FLQA-Wk" OR "wound assessment instrument*" OR "wound assessment tool*" OR "wound healing scale*" OR "wound classification" OR "wound measurement" OR "skin integrity assessment" OR validation OR psychometric* OR "content validity" OR reliab* OR "cross-cultural adaptation" OR "transcultural adaptation" OR "measurement propert*") AND ("Wounds" OR "Skin Lesions" OR "Cutaneous Injuries" OR "Tissue Damage" OR "Skin Ulcers" OR "Pressure Injuries" OR "Chronic Wounds" OR "Acute Wounds" OR "Traumatic Wounds" OR "Open Lesions" OR "Skin Breaks") AND ("Specialists" OR "Experts" OR "Healthcare Specialists" OR "Wound Care Experts" OR "Wound Specialists" OR "Clinical Specialists" OR "Subject Matter Experts" OR "Medical Specialists" OR "Professional Experts" OR "Consultants" OR "Specialized Clinicians") |
| Scopus | ("BWAT" OR "Bates-Jensen Wound Assessment Tool" OR "RESVECH" OR "RESVECH 2.0" OR "PUSH" OR "Pressure Ulcer Scale for Healing" OR "PUSH Tool" 3.0 OR "SINBAD" OR "DESIGN-R" OR "FLQA-Wk" OR "wound assessment instrument*" OR "wound assessment tool*" OR "wound healing scale*" OR "wound classification" OR "wound measurement" OR "skin integrity assessment" OR validation OR psychometric* OR "content validity" OR reliab* OR "cross-cultural adaptation" OR "transcultural adaptation" OR "measurement propert*")  AND (Wounds OR "Skin Lesions" OR "Cutaneous Injuries" OR "Tissue Damage" OR "Skin Ulcers" OR "Pressure Injuries" OR "Chronic Wounds" OR "Acute Wounds" OR "Traumatic Wounds" OR "Open Lesions" OR "Skin Breaks") AND (Specialists OR Experts OR "Healthcare Specialists" OR "Wound Care Experts" OR "Wound Specialists" OR "Clinical Specialists" OR "Subject Matter Experts" OR "Medical Specialists" OR "Professional Experts" OR Consultants OR "Specialized Clinicians") |
| Embase | ("BWAT" OR "Bates-Jensen Wound Assessment Tool" OR "RESVECH" OR "RESVECH 2.0" OR "PUSH" OR "Pressure Ulcer Scale for Healing" OR "PUSH Tool" 3.0 OR "SINBAD" OR "DESIGN-R" OR "FLQA-Wk" OR "wound assessment instrument*" OR "wound assessment tool*" OR "wound healing scale*" OR "wound classification" OR "wound measurement" OR "skin integrity assessment" OR validation OR psychometric* OR "content validity" OR reliab* OR "cross-cultural adaptation" OR "transcultural adaptation" OR "measurement propert*") AND (Wounds OR 'Skin Lesions' OR 'Cutaneous Injuries' OR 'Tissue Damage' OR 'Skin Ulcers' OR 'Pressure Injuries' OR 'Chronic Wounds' OR 'Acute Wounds' OR 'Traumatic Wounds' OR 'Open Lesions' OR 'Skin Breaks') AND (Specialists OR Experts OR 'Healthcare Specialists' OR 'Wound Care Experts' OR 'Wound Specialists' OR 'Clinical Specialists' OR 'Subject Matter Experts' OR 'Medical Specialists' OR 'Professional Experts' OR Consultants OR 'Specialized Clinicians') |
| Cochrane Library | ("BWAT" OR "Bates-Jensen Wound Assessment Tool" OR "RESVECH" OR "RESVECH 2.0" OR "PUSH" OR "Pressure Ulcer Scale for Healing" OR "PUSH Tool" 3.0 OR "SINBAD" OR "DESIGN-R" OR "FLQA-Wk" OR "wound assessment instrument*" OR "wound assessment tool*" OR "wound healing scale*" OR "wound classification" OR "wound measurement" OR "skin integrity assessment" OR validation OR psychometric* OR "content validity" OR reliab* OR "cross-cultural adaptation" OR "transcultural adaptation" OR "measurement propert*") AND (Wounds OR "Skin Lesions" OR "Cutaneous Injuries" OR "Tissue Damage" OR "Skin Ulcers" OR "Pressure Injuries" OR "Chronic Wounds" OR "Acute Wounds" OR "Traumatic Wounds" OR "Open Lesions" OR "Skin Breaks") AND (Specialists OR Experts OR "Healthcare Specialists" OR "Wound Care Experts" OR "Wound Specialists" OR "Clinical Specialists" OR "Subject Matter Experts" OR "Medical Specialists" OR "Professional Experts" OR Consultants OR "Specialized Clinicians") |
| Web of Science | ("BWAT" OR "Bates-Jensen Wound Assessment Tool" OR "RESVECH" OR "RESVECH 2.0" OR "PUSH" OR "Pressure Ulcer Scale for Healing" OR "PUSH Tool" 3.0 OR "SINBAD" OR "DESIGN-R" OR "FLQA-Wk" OR "wound assessment instrument*" OR "wound assessment tool*" OR "wound healing scale*" OR "wound classification" OR "wound measurement" OR "skin integrity assessment" OR validation OR psychometric* OR "content validity" OR reliab* OR "cross-cultural adaptation" OR "transcultural adaptation" OR "measurement propert*") AND ("Wounds" OR "Skin Lesions" OR "Cutaneous Injuries" OR "Tissue Damage" OR "Skin Ulcers" OR "Pressure Injuries" OR "Chronic Wounds" OR "Acute Wounds" OR "Traumatic Wounds" OR "Open Lesions" OR "Skin Breaks") AND ("Specialists" OR "Experts" OR "Healthcare Specialists" OR "Wound Care Experts" OR "Wound Specialists" OR "Clinical Specialists" OR "Subject Matter Experts" OR "Medical Specialists" OR "Professional Experts" OR "Consultants" OR "Specialized Clinicians") |
| LILACS / SciELO / BDENF (way BIREME — iAHx or VHL) | ("BWAT" OR "Bates-Jensen" OR "RESVECH" OR "PUSH" OR "SINBAD" OR "DESIGN-R" OR "FLQA-Wk" OR "instrumento de avaliação" OR "escala de avaliação" OR "instrumento de medida" OR "escala de feridas" OR "classificação de feridas" OR "avaliação de feridas" OR validação OR "validade de conteúdo" OR "estudo de validação" OR psicometria OR "propriedades psicométricas" OR confiabilidade OR "adaptação transcultural" OR "propriedades de medida") [Palavras] AND (feridas OR "lesão de pele" OR "lesão cutânea" OR "lesão de tecido" OR úlcera OR "úlcera por pressão" OR "lesão por pressão" OR "ferida crônica" OR "ferida aguda" OR "ferida traumática" OR "pé diabético" OR "úlcera venosa" OR "úlcera arterial" OR "ferida cirúrgica") [Palavras] AND (especialistas OR experts OR "juízes" OR validadores OR "profissionais especializados" OR "painel de especialistas" OR "comitê de juízes" OR "consultores" OR "avaliadores") [Palavras] |
| Additional strategy using DeCS descriptors (for controlled search): | (mh:"Avaliação em Enfermagem" OR mh:"Estudos de Validação" OR mh:"Psicometria" OR mh:"Reprodutibilidade dos Testes" OR mh:"Comparação Transcultural" OR "validade de conteúdo" OR "adaptação transcultural") AND (mh:"Ferimentos e Lesões" OR mh:"Úlcera por Pressão" OR mh:"Úlcera Varicosa" OR mh:"Pé Diabético" OR mh:"Cicatrização de Feridas" OR "ferida crônica") AND (mh:"Competência Clínica" OR mh:"Papel do Profissional de Enfermagem" OR especialistas OR "comitê de especialistas" OR "painel de especialistas") |
