# Supplementary material for: Validation of Wound Assessment Instruments Across Brazilian Clinical Contexts: Protocol for a Scoping Review
Source: JMIR Res Protoc. 2026 Aug 24;15:e99483. doi: 10.2196/99483 (PMC13502934; doi:10.2196/99483)
Supplement: Supplementary file 2 [file resprot-v15-e99483-s002.docx]

## Multimedia Appendix 2: Data Extraction Instrument – Scoping Review

Review title: Validation of wound assessment instruments across Brazilian clinical contexts: a scoping review

**Table 3. Unified data extraction instrument.**

| **Domain** | **Variable** | **Description / Data Collected** |
| --- | --- | --- |
| **1. Source Identification** | Study ID / code | Sequential number or unique identifier |
|  | Author(s) | Name(s) of the main author(s) |
|  | Year of publication | Year in which the study was published |
|  | Study title | Full title as published |
|  | Source / Journal | Name of the journal or publication source |
|  | DOI / Link | Direct link or DOI |
|  | Country of origin | Location where the study was conducted or published |
|  | Publication type | Scientific article, thesis, dissertation, technical report, guideline, etc. |
|  | Funding / Conflict of interest | Is funding declared? Is a conflict of interest reported? |
| **2. Source Characteristics** | Study objective | Primary objective of the publication |
|  | Study type / methodology | Quantitative, qualitative, mixed-methods, review, validation study, etc. |
|  | Participants | Professionals, patients, experts? Number and characteristics |
|  | Clinical context | Hospital, primary care, outpatient, home care, other |
|  | Target population of instrument | Adults, older adults, patients with chronic wounds, ostomy patients, etc. |
|  | Instrument assessed | Name of the wound assessment instrument |
|  | Validation stages reported | Content validity; Criterion validity; Reliability; Responsiveness/Sensitivity |
|  | Type of analysis conducted | Statistics used (e.g., Cronbach's alpha, kappa, ICC, etc.) |
|  | Validation framework used | Methodological framework employed in the validation (e.g., COSMIN, Beaton, Pasquali, Polit & Beck) |
|  | Number of experts | Total number of expert judges/validators who participated in the validation |
|  | Expert eligibility criteria reported | Were eligibility criteria for expert selection explicitly reported? (Yes / No / Partially) |
|  | Expert involvement | Yes/No – Number and profile of experts involved |
| **3. Results and Evidence** | Main results | Key findings regarding validity, reliability, and applicability |
|  | Psychometric indicators | Quantitative values for reliability, validity, responsiveness, etc. |
|  | Identified barriers | Difficulties in application, adaptation, acceptance, or standardization |
|  | Improvement suggestions | Adjustments proposed for the instrument |
|  | Applicability across contexts | Was the instrument tested in more than one clinical setting? Which ones? |
|  | Cultural/linguistic adaptation | Was transcultural validation performed? Was it translated into Portuguese? |
| **4. Identified Gaps** | Lack of robust validation | Yes/No – Are there clear methodological limitations? |
|  | Lack of expert assessment | Yes/No – Were experts consulted? |
|  | Underrepresented clinical settings | Primary care, rural settings, university hospital, basic health unit (UBS), etc. |
|  | Limitations reported by authors | Was a critical self-appraisal presented? What were the limitations? |
| **5. Additional Notes** | General comments | Reviewer notes (insights, connections with other studies, limitations, etc.) |
|  | Relevance to review objective | High / Medium / Low |
|  | Additional data to explore | Aspects not pre-specified but relevant to the analysis |

Note: This instrument may be implemented as an Excel spreadsheet, a Google Form, or directly within review software (e.g., JBI SUMARI, Covidence, Rayyan), with editable fields and space for comments.
